# Overexpression of KIAA1199, a novel strong hyaluronidase, is a poor prognostic factor in patients with osteosarcoma

**DOI:** 10.1186/s13018-021-02590-4

**Published:** 2021-07-07

**Authors:** Kan Ito, Yoshihiro Nishida, Kunihiro Ikuta, Hiroshi Urakawa, Hiroshi Koike, Tomohisa Sakai, Jiarui Zhang, Yoshie Shimoyama, Shiro Imagama

**Affiliations:** 1 Department of Orthopedic Surgery, Nagoya University Graduate School of Medicine, 65 Tsurumai, Showa, Nagoya, Aichi 466-8550 Japan; 2 Department of Rehabilitation, Nagoya University Hospital, 65 Tsurumai, Showa, Nagoya, Aichi 466-8550 Japan; 3 Department of Pathology and Laboratory Medicine, Nagoya University Hospital, 65 Tsurumai, Showa, Nagoya, Aichi 466-8550 Japan

**Keywords:** Osteosarcoma, KIAA1199, Hyaluronan, Prognostic factor, Immunostaining

## Abstract

**Background:**

Hyaluronan (HA) has been shown to play important roles in the growth, invasion, and metastasis of malignant tumors. KIAA1199, which has potent HA-degrading activity, has been reported to be expressed in various malignancies and associated with patient prognosis. However, there are no reports on the expression of KIAA1199 in osteosarcoma. The aim of this study was to investigate the impact of KIAA1199 and HA expression in osteosarcoma tissues on the prognosis and other clinical characteristics of osteosarcoma patients.

**Methods:**

From 2003 to 2013, we included 49 patients with osteosarcoma at our institution, whose FFPE (formalin fixed paraffin embedded) tissue was available at the time of biopsy. The expressions of KIAA1199 and HA in each sample were assessed by immunohistochemistry using the primary antibody for KIAA1199 and HA-binding protein (HABP), respectively. For evaluation of the positivity of KIAA1199 staining, we divided the samples into two groups: High group with more than 75% positive staining and Low group with less than 75% positive staining. In the HABP staining, those with more than and less than 60% were assigned to a High group, and Low group respectively. Various clinical features were correlated with staining positivity. Prognostic factors including positivity of the staining were analyzed. Levels of mRNA expression for enzymes related to HA metabolism were assessed in two osteosarcoma cell lines using real-time RT-PCR.

**Results:**

In KIAA1199 staining, high positivity was significantly correlated with occurrence of distant metastases (P = 0.002). The necrosis rate after preoperative chemotherapy was significantly lower in the High positivity group (59%), compared to that in the Low group (84.8%) (P = 0.003). HABP positivity was not correlated with any demographic variables, although the Low positivity group had a significantly better overall survival than the High group with KIAA1199 and HABP staining (P = 0.026 and P = 0.029, respectively). In multivariable analysis, KIAA1199 (P = 0.036) and HABP staining (P = 0.002), location (P = 0.001), and distant metastasis at initial diagnosis (P < 0.001) were identified as significant prognostic factors. KIAA1199 and hyaluronan synthase mRNA were expressed at different levels in the two osteosarcoma cell lines.

**Conclusions:**

Our results showed that high expression of KIAA1199 and HA are both poor prognostic factors in osteosarcoma. KIAA1199 may be a useful marker for distant metastasis and chemoresistance.

## Background

Osteosarcoma is the most common primary malignant bone tumor in the pediatric and adolescent population [1]. Its annual incidence is not high, approximately 2-3.4 people per million [2, 3]. Over 90% of osteosarcomas are histopathologically classified as high grade malignancies [4]. The 5-year overall survival rate for patients with osteosarcoma is 65-76% for non-metastatic patients [5, 6] but is as low as 19-29% for metastatic ones [7, 8]. Approximately, 40% of patients initially diagnosed with localized osteosarcoma relapse after surgery, particularly in the lung [9], indicating the crucial need for identification of prognostic factors for the prediction of distant metastasis. The standard treatment strategy for localized osteosarcoma is wide resection of the tumor combined with multi-agent adjuvant chemotherapy before and after surgery. The three effective agents for osteosarcoma are cisplatin, doxorubicin, and high-dose methotrexate. Combinations of these drugs administered pre-and post-operatively have been widely used. The most common prognostic factors are distant metastases, histologic response to preoperative chemotherapy, and complete surgical resection [5, 6, 10].

Hyaluronan (HA) is a high molecular weight glycosaminoglycan that is one of the main components of the extracellular matrix (ECM). HA is known to play important roles in matrix assembly, cell proliferation, differentiation, and migration during development, normal tissue homeostasis, and disease [11]. HA synthesized by HAS2 has been reported to play crucial roles in cell proliferation, migration, and invasiveness in osteosarcoma cell lines [12]. However, no clinical studies have analyzed the correlation between HA expression levels and clinical features of osteosarcoma.

KIAA1199 is the first gene identified in association with non-syndromic hearing loss [13] and has recently become known as a strong HA-degrading enzyme [14]. Upregulation of KIAA1199 expression is associated with cancer progression and has been reported to predict poor prognosis in various cancers, including colorectal cancer [15], gastric cancer [16], breast cancer [17], non-small cell lung cancer [18], pancreatic cancer [19], hepatocellular carcinoma [20], ovarian cancer [21], and papillary thyroid cancer [22]. Several authors have described the association of KIAA1199 with cell proliferation, motility, and apoptosis [17, 23]. But no such studies have analyzed its association with the prognosis of patients with osteosarcoma.

The aim of this study is to investigate the relationship between the expression levels of KIAA1199 in addition to HA, which could be detected by HA-binding protein (HABP) with immunostaining for osteosarcoma tissues, and their correlation with various clinical characteristics and patients’ prognosis.

## Methods

### Patient eligibility and characteristics

Between January 2003 and December 2013, 54 patients with osteosarcoma were treated in our institution. Five of them were excluded because of the lack of clinical information and/or biopsy specimens. Forty-nine patients with osteosarcoma, for whom FFPE (formalin fixed paraffin embedded) tissues were available at the time of biopsy, were enrolled in this study. Biopsy specimens unexposed to chemotherapeutic agents were subjected to the analyses. All diagnoses were confirmed by an experienced pathologist (Y. S.). Clinical information including age, gender, tumor size, tumor location, histology, distant metastasis, staging, tumor necrosis rate after pre-operative chemotherapy, and prognostic status was reviewed from the patients’ medical records. Disease staging was determined according to the AJCC (The American Joint Committee on Cancer) classification system, 8th edition [24]. Forty-five patients received wide resection and peri-operative chemotherapy, and four (three pelvic and one lower extremity) were not treated with surgery. None of the three pelvic patients received surgery, but were treated with chemotherapy. In addition, two of them received heavy ion radiotherapy, and one conventional radiotherapy. One patient with lower extremity was treated with chemotherapy and conventional radiotherapy. Among 49 patients, 37 patients received MAP (Methotrexate, Doxorubicin, Cisplatin) or MAP-I (Methotrexate, Doxorubicin, Cisplatin, Ifosfamide) chemotherapy, 4 received AI (Doxorubicin, Ifosfamide), 3 received other regimens, and 5 had no information. Tumor necrosis rate could not be identified in nine of the patients because four were not indicated for surgery, two did not receive preoperative chemotherapy, and three had surgery at other hospitals. Follow-up period was defined as the duration between first visit to our institution and last visit or the time of patient death. The average follow-up period was 84 months (range, 1-202 months). The clinicopathological characteristics of these patients are shown in Table 1. Correlation of the factors with stainability of KIAA1199 and HA was analyzed. Prognostic factors for local recurrence-free survival and overall survival were determined including positivity of KIAA1199 and HABP. This study was approved by the Institutional Review Board in our institution (Approval No.1332), and conducted in compliance with the Declaration of Helsinki. Informed consent was waived because of the retrospective nature of the study and met the exemption requirements in the ethical guidelines for epidemiological research.

**Table 1:** Patient characteristics between clinicopathological variables and stainability of KIAA1199 and HABP

		KIAA1199			HABP		
	All patients (n = 49)	High (n = 34)	Low (n = 15)	P value	High (n = 21)	Low (n = 28)	P value
Gender	Male 30 Female 19	Male 21 Female 13	Male 9 Female 6	0.91	Male 11 Female 10	Male 19 Female 9	0.27
Age (years)	23.1 (7-74)	24.3	20.3	0.49	24.1	22.3	0.75
Size (cm)	11.5 (4-26)	11.1	12.2	0.57	12.8	10.5	0.16
Location				0.91			0.76
Pelvis	3	2	1		2	1	
Humerus	3	2	1		1	2	
Femur	19	12	7		9	10	
Tibia	23	17	6		9	14	
Trunk	1	1	0		0	1	
Histology				0.51			0.17
Osteoblastic	23	17	6		7	16	
Fibroblastic	13	7	6		6	7	
Chondroblastic	12	9	3		8	4	
Telangiectatic	1	1	0		0	1	
Metastasis							
All	27	24	3	0.002	13	14	0.3
Exists at initial diagnosis	10	9	1	0.11	3	7	0.36
AJCC classification (8th edition)				0.21			0.79
IIA	14	9	5		4	10	
IIB	24	16	8		13	11	
IV	10	9	1		3	7	
Unknown	1	0	1		1	0	
Necrosis rate (%)	67.4 (15-100) (unknown 9)	59	84.8	0.003	77.5	61.2	0.09
HABP positivity (%)	61.7 (14.4-99.6)	61.7	61.8	0.99	―	―	―
KIAA1199 positivity (%)	76.8 (16.2-97.3)	―	―	―	75.3	77.9	0.65

### Cell lines

Two human osteosarcoma cell lines, HOS and Saos-2, were used in this study. These cell lines were a kind gift from Dr. Akihiko Takeuchi (Kanazawa University, Ishikawa Japan). These cell lines were grown in a humidified atmosphere at 37 °C with 5% CO_2_ in DMEM medium supplemented with 10% fetal bovine serum (FBS), 100 units/ml penicillin, and 100 mg/ml streptomycin.

### Immunohistochemistry

All tumor samples were obtained by incisional biopsy in advance of pre-operative chemotherapy. Tumor sections cut to 4 μm thickness were subjected to the staining for KIAA1199 and HA. Next, the specimens were treated with the corresponding antibodies at 4 °C overnight in a moist chamber: polyclonal rabbit anti-KIAA1199 antibody (21129–1-AP; Proteintech Group, Inc., Chicago, IL, USA; diluted 1:200) and biotinylated HA-binding protein (b-HABP; Hokudo, Sapporo, Japan; diluted 1:250). When staining with b-HABP, prior to the inactivation of endogenous peroxidase, sections were incubated with chondroitinase ABC (0.25 units/ml, pH 8.0) at 37 °C for 60 min. Stained sections incubated without primary antibodies were used as negative controls.

Breast cancer tissues were used as a positive control for KIAA1199 staining [17], and normal colon tissues, which are reported to show almost no expression of KIAA1199 [15, 25], were used as a negative control.

### Evaluation of stainability

Three fields (magnification ×400) were randomly selected in each section. KIAA1199-positive cells or HABP-positive cells in these fields were counted, and divided by the total number of tumor cells in each field. For positivity of KIAA1199 staining, we divided the patients into two groups, High group (≧ 75% positive cells) and Low group (< 75% positive cells). Similarly, in positivity of HABP staining, the patients with > 60% positive cells were defined as High group and those with < 60% were defined as Low group. These cutoff values, 75% and 60% for KIAA1199 and HABP, respectively, were determined by C-index as the most sensitive values for overall survival (OS).

All slides were evaluated independently by two blinded observers (KI and HK). A joint analysis by the two observers was performed where discrepancies were observed to reach a consensus.

### Real-time quantitative RT-PCR

Expression levels of mRNA for HAS1, HAS2, HAS3, HYAL1, HYAL2, KIAA1199, and GAPDH were determined in two osteosarcoma cell lines, HOS and Saos-2. Total cellular RNA was isolated using RNeasy Mini Kit (Qiagen, Hilden, Germany), according to the manufacturer’s instructions. Following conventional reverse transcriptase-polymerase chain reaction (RT-PCR), cDNA was subjected to real-time RT-PCR for semi-quantification of mRNAs for each enzyme using a LightCycler (Roche Diagnostics). The relative levels of mRNA were expressed as relative quantification normalized with expression levels of GAPDH mRNA. Each enzyme’s primer pairs are shown in Table 2.

**Table 2:** List of genes and their specific primer sequences

Gene name	Primer	Sequence
HAS1	Forward	5′-CAGACCCACTGCGATGAGAC-3′
	Reverse	5′-CCACCAGGTGCGCTGAAA-3′
HAS2	Forward	5′-TCAGAGCACTGGGACGAAG-3′
	Reverse	5′-CCCAACACCTCCAACCAT-3′
HAS3	Forward	5′-CAGCAACTTCCAATGAGGC-3′
	Reverse	5′-CACAGTGTCAGAGTCGCA-3′
HYAL1	Forward	5′-GCAGTAGCCCAGGACCAGTT-3′
	Reverse	5′-GCAGTCAGGGAAGCCATAGA-3′
HYAL2	Forward	5′-CTGCCCTGATGTTGAGGTG-3′
	Reverse	5′-GGAGGAAGCAAGTGTCTCGT-3′
KIAA1199	Forward	5′-AGACTAGCTACCACTCCGCT-3′
	Reverse	5′-TCAGCATGGCCTTGAAGAGG-3′
GAPDH	Forward	5′-TGAACGGGAAGCTCACTGG-3′
	Reverse	5′-TCCACCACCCTGTTGCTGTA-3′

### Statistical analysis

We determined the optimal cutoffs for KIAA1199 and HABP staining to divide the patients into two groups, High and Low groups. We created 10,000 candidate cutoffs by dividing the KIAA1199 positivity evenly between 0 and 100%. For each candidate cutoffs, we performed univariate Cox proportional hazard models for OS. A line graph was then created with each cutoff on the horizontal axis and the C-index [26], which indicates the predictive ability of the model, on the vertical axis. The threshold value with the maximum C-index was determined to be the optimal cutoff. The optimal cutoff for HABP was calculated by using the same method.

Interobserver reliability in categorizing into two groups, the High group and the Low group, in immunostaining was evaluated using Cohen’s kappa. To evaluate the relationship between clinicopathological features and immunohistochemical positivity, Pearson’s chi-square test was used for categorical variables, and Mann-Whitney’s U test for continuous variables. OS period and disease-free survival (DFS) period were calculated from the date of the first visit to that of death or final follow-up, or the first event recorded (local recurrence or metastasis), respectively. For DFS, 12 patients with distant metastasis at the first visit or non-operative patients, and one patient with insufficient information were excluded, resulting in 36 patients being included in the analysis. Survivorship was determined with the Kaplan-Meier method, and the association between patient prognosis and various factors including the positivity of immunostaining was assessed by the log-rank test. Factors with P value < 0.05 in the univariate analysis by log-rank test were subjected to multivariable analysis using a Cox proportional hazard model. Because of small number of patients, the Cox proportional hazard model was corrected by Firth’s method [27]. Procedure for calculating the optimal cutoff and multivariable analysis was conducted using R (R Development Core Team 2021), while all other analyses were conducted using SPSS 27 (IBM Corporation, Armonk, NY, USA). In all analyses, P values < 0.05 were considered statistically significant.

## Results

### Immunohistochemical staining with anti-KIAA1199 polyclonal antibodies or b-HABP in osteosarcoma tissues

**Fig. 1:**
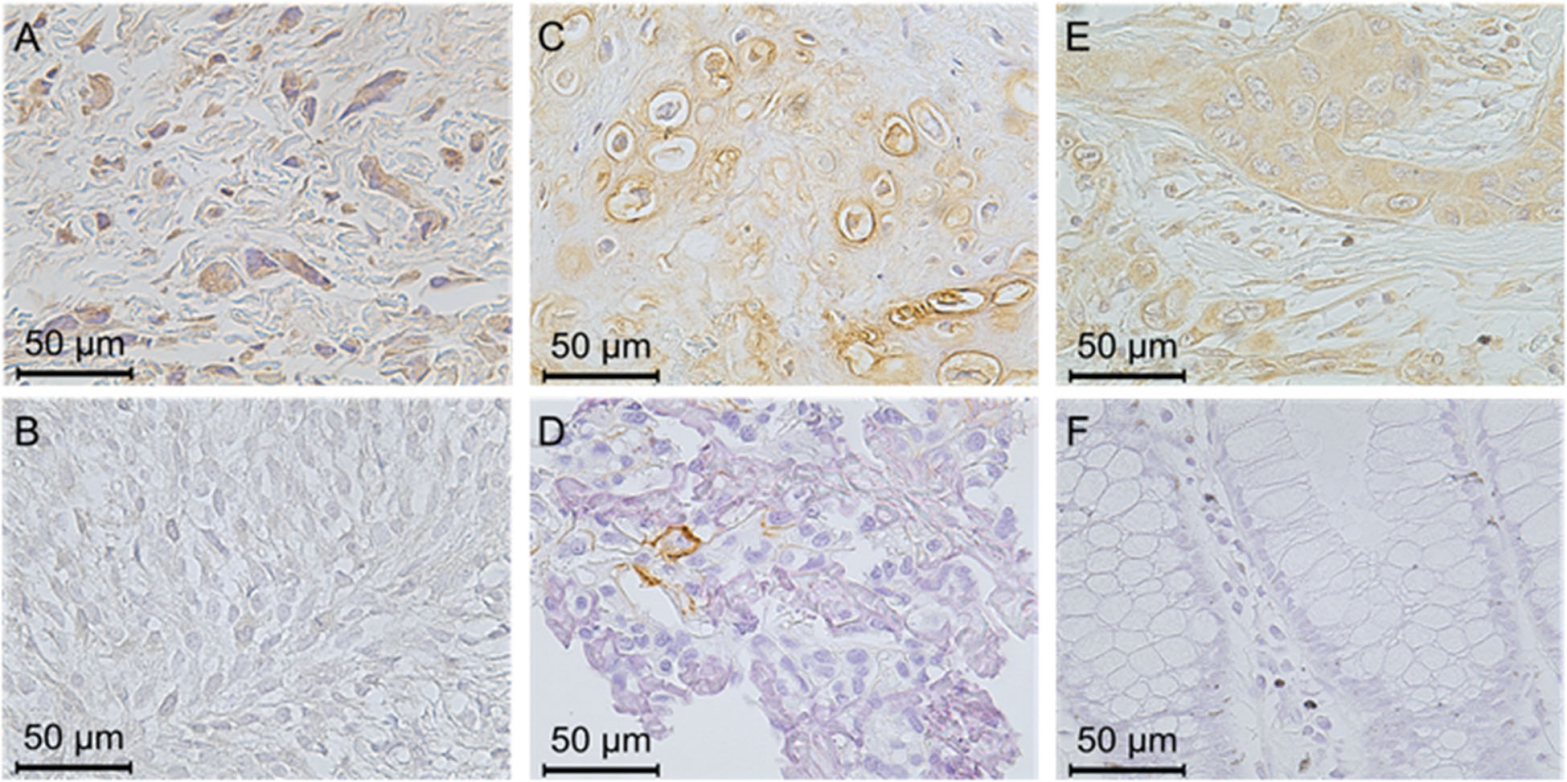
Immunohistochemical staining for KIAA1199 and HABP. (**A**) High KIAA1199 positivity in osteosarcoma. (**B**) Low KIAA1199 positivity in osteosarcoma. (**C**) High HABP positivity in osteosarcoma. (**D**) Low HABP positivity in osteosarcoma. (**E**) Staining for KIAA1199 in breast cancer. (**F**) Staining for KIAA1199 in normal colon tissue

KIAA1199 and HA expressions in osteosarcoma tissues obtained from the 49 patients were measured by immunostaining. KIAA 1199 was localized principally in tumor cell cytoplasm, and HA in cytoplasmic/nuclear and extracellular areas. We evaluated the positivity focusing on the cytoplasmic staining for KIAA1199 and cytoplasmic/nuclear staining for HA. Figure 1 A, B, C, and D are typical pictures of osteosarcoma tissues immunostained with anti-KIAA1199 polyclonal antibodies or b-HABP, showing high and low positivity. Figure 1 E and F show breast cancer tissue as a positive control and normal colon tissue as a negative control on KIAA 1199 staining, respectively.

**Fig. 2:**
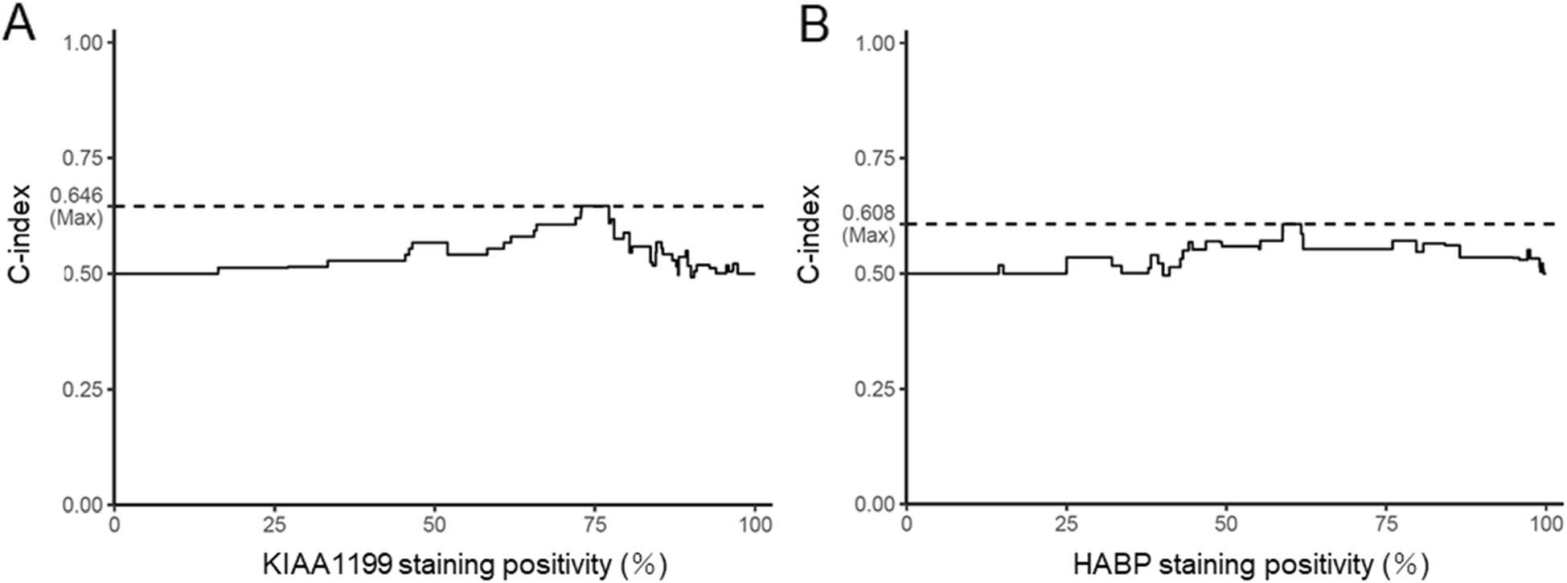
Graphs of C-index. (**A**) KIAA1199 staining; (**B**) HABP staining

The C-index took a maximum value of 0.646 when the staining positivity of KIAA1199 was 72.90-77.19%. As a representative of the range, 75% was determined as the cut-off value (Fig. 2A). In the same way, the C-index took a maximum value of 0.608 when the staining positivity of HABP was 58.80-61.69%, and as representative of this range, 60% was determined as the cutoff value (Fig. 2B).

### Comparison between the positivity of KIAA1199 or HABP staining and clinicopathological variables in osteosarcoma patients

The characteristics of all 49 patients are shown in Table 1. There were 30 males and 19 females, the mean age at biopsy was 23 years (range 7-74 years), and the mean tumor size was 11.5 cm (range 4-26 cm). The tumor sites were extremities in 45 patients, pelvis in 3, and sternum in one. The histological type was osteoblastic in 23 patients. Twenty-seven patients had distant metastasis, and were present in ten at the time of the initial diagnosis. Status of metastasis was unknown in one of the 49 patients, who were in the Low group in KIAA1199 staining and in the High group in HABP staining. In the evaluation of KIAA1199 staining, 34 patients were categorized into the High group and 15 into the Low group. In HABP staining, 21 patients in the High group and 28 in the Low group. Interobserver agreement for categorizing was 91.8% (Cohen’s kappa = 0.80 and SE = 0.095) for KIAA1199 staining, 89.8% (Cohen’s kappa = 0.795 and SE = 0.086) for HABP staining. The associations between the positivity of the staining and clinicopathological variables are shown in Table 1.

In the evaluation of KIAA1199 staining, distant metastases were significantly more frequent in the High group compared with the Low group (P = 0.002). Among the 38 patients with localized disease at the initial diagnosis, distant metastases also occurred significantly more frequently in the High group (P = 0.009). In addition, the necrosis rate evaluated in resected tumor tissue after pre-operative chemotherapy was significantly lower in the High group compared to that in the Low group (P = 0.003). On the other hand, HABP staining showed no significant difference between the two groups in any of the clinicopathological variables.

### Survival assessment

**Fig. 3:**
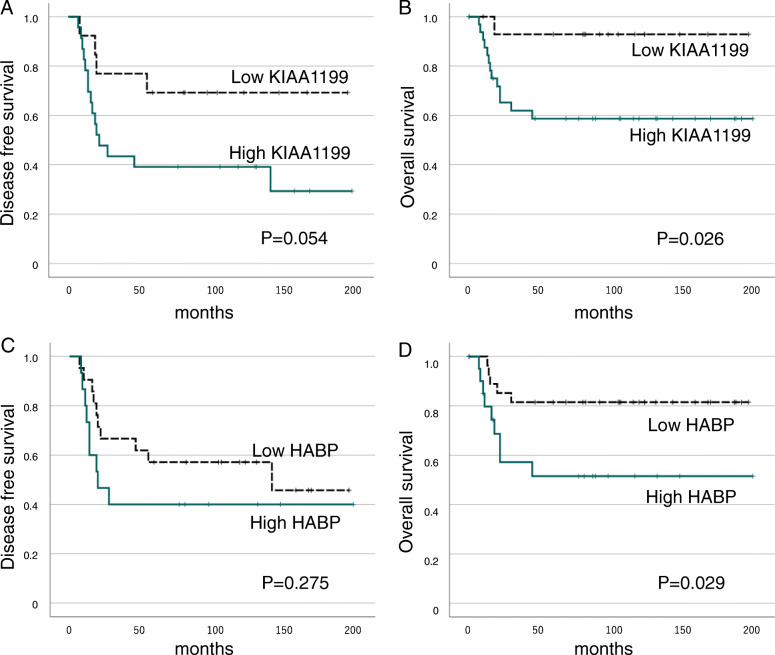
Kaplan-Meier analysis of in patients with osteosarcoma according to KIAA1199 and HABP staining. DFS (**A**) and OS (**B**) according to KIAA1199 positivity. Rigid and dotted lines indicate High and Low KIAA1199 positivity group, respectively. (**A**: n = 36, **B**: n = 49). DFS (**C**) and OS (**D**) according to HABP positivity. Rigid and dotted lines indicate High and Low HABP positivity group, respectively (**C**: n = 36, **D**: n = 49)

The results of survival analysis in KIAA staining showed that the 5-year DFS was 69.2% in the Low group and 39.1% in the High group (Fig. 3A), and the 5-year OS was 92.9% in the Low group and 58.7% in the High group (Fig. 3B). Although there was no significant difference between the two groups in DFS, the Low group tended to have a longer DFS than the High group (P = 0.054). In OS, the Low group had a significantly better survival than the High group (P = 0.026).

In HABP staining, 5-year DFS was 57.1% in the Low group and 40.0% in the High group (Fig. 3C), and 5-year OS was 81.5% in the Low group and 51.5% in the High group (Fig. 3D). There was no significant difference in DFS (P = 0.275), whereas OS in the Low group was significantly better compared with that in the High group (P = 0.029).

### Univariate and multivariable analyses for factors affecting survival in patients with osteosarcoma

The results of the analysis are shown in Table 3. In the univariate analysis, no factor significantly contributed to DFS, while pelvis location, distant metastasis, high positivity in KIAA1199 staining, and high positivity in HABP staining were identified as significant poor prognostic factors in OS (P = 0.018, P = 0.007, P = 0.026, P = 0.029, respectively). In multivariable analysis, these four factors also showed significant differences, indicating that they are independent prognostic factors for OS (P = 0.001, P < 0.001, P = 0.036, P = 0.002, respectively).

**Table 3:** Univariate and multivariable analysis of factors associated with DFS and OS

Parameter	DFS n = 36	OS n = 49		
	P value			P value
Univariate analysis*				
Gender (male/female)	0.137			0.623
Age (60 ≦/< 60 years)	0.747			0.945
Size (8 ≦/< 8 cm)	0.706			0.107
Location (pelvis/others)	0.685			0.018
Histology (osteoblastic/others)	0.524			0.511
Metastasis (present/absent)	―			0.007
Necrosis rate (90 ≦/< 90%)	0.495			0.458
KIAA1199 positivity (high/low)	0.054			0.026
HABP positivity (high/low)	0.275			0.029
Multivariable analysis♱		HR	95% CI	P value
Location (pelvis/others)	―	39.99	5.16-358.96	0.001
Metastasis (present/absent)	―	15.98	3.38-101.47	< 0.001
KIAA1199 positivity (high/low)	―	5.08	1.10-51.65	0.036
HABP positivity (high/low)	―	8.62	2.08-52.13	0.002

*Log-rank test, ♱Cox regression model with Firth’s correction method

### mRNA expression of enzymes related to HA metabolism in osteosarcoma cell lines

**Fig. 4:**
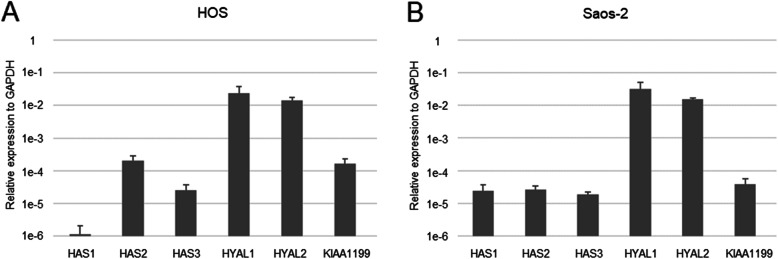
Expression levels of mRNA for enzymes related to HA metabolism in osteosarcoma cell lines. The mRNA expression levels were determined by real-time RT-PCR and presented as relative values normalized with that of GAPDH. (**A**) expression in HOS; (**B**) expression in Saos-2

Figure 4 shows expression levels of each mRNA for enzymes related to HA metabolism in two different osteosarcoma cell lines, HOS and Saos-2. The expression pattern of HYAL1 and HYAL2 showed similar proportions in the two cell lines. In contrast, the expression levels of HAS1-3 and KIAA1199 differed between them.

## Discussion

The present study is the first report to investigate the association between positivity of KIAA1199 and HA expression in immunohistochemistry and prognosis and various clinicopathological features of patients with osteosarcoma. We found that high expression of KIAA1199 was significantly associated with distant metastasis, tumor necrosis rate, and decreased overall survival of osteosarcoma. High expression of HA was also associated with decreased overall survival in osteosarcoma. Previous studies have found KIAA1199 to be overexpressed in a variety of malignancies [15–22, 28]. Of these reports, in all cancers, except oral squamous cell carcinoma, KIAA1199 has been reported to be associated with patient prognosis. In osteosarcoma, like these other malignancies, KIAA1199 expression was found to be a prognostic factor.

KIAA1199, also referred to as cell migration inducing protein (CEMIP), plays a role in the development and maintenance of cancer metastasis. Wang et al. researched the function of KIAA1199 in gastric cancer and reported that the introduction of *KIAA1199* both in vitro and in vivo dramatically promoted the proliferation and migration of tumor cells, while its inhibition suppressed them and induced apoptosis of tumor cells [29]. Other studies also reported that the tumorigenicity of malignant tumor cells was suppressed by knockdown of KIAA1199 in gastric cancer and other cancers [16, 17, 30]. The conventional or telangiectatic osteosarcomas that were the subject of this study are generally high grade tumors, and the results of this study suggested that overexpression of KIAA1199 might contribute to the promotion of tumorigenicity, as reported in previous high-grade cancers.

Common prognostic factors for osteosarcoma have been reported to be tumor site, size, distant metastasis, response to chemotherapy, and complete resection [10]. In this study, multivariable analysis also identified tumor site and distant metastasis as prognostic factors. Because the prognostic factors identified in the present study were similar to those in previous reports, the population of this study was considered to be suitable for the analysis.

In this study, distant metastasis and necrosis rate were significantly correlated with the positivity of KIAA1199 staining. This result suggests that KIAA1199 can be a possible marker for prediction of distant metastasis and necrosis rate. The necrosis rate was not identified as a prognostic factor in the univariate analysis in our results, unlike the results of previous reports [5, 6, 10]. This could be because the analysis was based on a small number of patients and had not enough power to detect the difference. In previous reports, necrosis rate was not identified as a prognostic factor in a small analysis of 29 pediatric osteosarcoma patients with pulmonary metastases at initial diagnosis [31], or in an analysis of 28 osteosarcoma patients older than 40 years [32].

There was not a sufficiently significant difference in DFS between the High and Low groups (P = 0.054) in the present study. This may be attributable to the fact that only 36 patients were analyzed, excluding patients with distant metastasis at initial diagnosis and non-surgical patients. If the number of patients had been greater, a significant difference might have been found in DFS between the two groups. In the evaluation of HABP staining, there was no significant difference in DFS between the High and Low groups (P = 0.275), or in the incidence of metastasis between the two groups. These results suggest that the metastatic ability of osteosarcoma is more likely associated with high expression of KIAA1199 than that of HA.

HA is produced by three HA synthases (HAS1, HAS2, and HAS3) at the intracellular border of the plasma membrane and extruded to the cell surface and extracellular matrix [33]. Previous studies have described that extracellular HA stimulates growth, migration, and invasion of various malignancies [34, 35]. Besides, elevated HA levels in tumor tissue correlate with poor prognosis in patients with malignancies such as ovarian, lung, thyroid, breast, colorectal, and gastric cancer [36–41]. Regarding sarcomas, a previous report demonstrated that high expression of HA was a poor prognostic factor in malignant peripheral nerve sheath tumor [42]. In relation to osteosarcoma, HAS2-mediated HA synthesis has been reported to play an important role in cell proliferation, migration, and invasiveness in the osteosarcoma cell line MG63 [12]. Tofuku et al. reported that HAS3-related HA enhances metastatic potential in the osteosarcoma cell line LM8 [43]. On the other hand, the detailed mechanism of HA degradation remains unclear. It has been reported that the HA receptor CD44 and two HA-degrading enzymes (HYAL1 and HYAL2) work together [44]. HA-degrading activity of HYAL1 and 2 was very weak, and KIAA 1199 has been shown to be a novel strong HA-degrading enzyme, having a completely different pathway of HA degradation [14]. In the present study, KIAA1199 was overexpressed in patients with osteosarcoma with a poor prognosis. This may be a response to the abundantly produced HA in osteosarcoma with a poor prognosis.

In the present study, we also observed the mRNA expression of HA metabolism-related enzymes in the human osteosarcoma cell lines, HOS and Saos-2. The expression levels of HAS1-3 and KIAA1199 were different between these cell lines. These results suggest that HA metabolism actively occurs in osteosarcoma cells and that the pattern of metabolism varies among cells, probably reflecting individual differences. A previous report demonstrated that cell growth of HOS cells was much faster than that of Saos-2 cells, while gene expression of VEGF was sixfold higher in Saos-2 cells than that in HOS cells [45]. Since the expression of various molecules in osteosarcoma is expected to differ depending on the cases, it will be necessary in the future to clarify the roles played by HA-related enzymes and the mechanism in tumorigenicity.

The reciprocal nature of the expression of KIAA1199 and HA is unknown. The results of this study did not show a meaningful correlation between KIAA1199 and HAS expression. It should be further analyzed whether KIAA1199 and HAS expressions are correlated or not.

There are several limitations to this study. First, the number of patients included was small. Second, due to the retrospective study design, there was a lack of detailed patient information for some patients, particularly the necrosis rate in nine patients. Third, the precise mechanism of KIAA1199 involvement in the tumorigenicity of osteosarcoma was not clarified.

## Conclusions

This study suggests that KIAA1199, which has strong HA degradation activity, may have the potential to predict chemoresistance and distant metastasis. In addition, high expressions of KIAA1199 and HA were both shown to be poor prognostic factors for osteosarcoma. Since these expressions are relatively easy to assess by immunostaining, they may be clinically useful in assessing the prognosis of osteosarcoma patients. Furthermore, KIAA1199 might be a novel therapeutic target in osteosarcoma.

## Data Availability

The datasets used and/or analyzed during the current study are available from the corresponding author on reasonable request.

## Abbreviations

AJCC: American Joint Committee on Cancer
b-HABP: Biotinylated hyaluronic acid-binding protein
CI: Confidence interval
C-index: Concordance index
DMEM: Dulbecco’s modified eagle medium
DFS: Disease-free survival
FFPE: Formalin fixed paraffin embedded
HA: Hyaluronan
HABP: HA-binding protein
HAS: Hyaluronan synthase
HYAL: Hyaluronidase
HR: Hazard ratio
OS: Overall survival
RT-PCR: Reverse transcriptase-polymerase chain reaction
